# The effects of inhibition and siRNA knockdown of collagen-binding integrins on human umbilical vein endothelial cell migration and tube formation

**DOI:** 10.1038/s41598-022-25937-1

**Published:** 2022-12-14

**Authors:** Emma J. Hunter, Samir W. Hamaia, Peter S.-K. Kim, Jean-Daniel M. Malcor, Richard W. Farndale

**Affiliations:** 1 Department of Biochemistry, University of Cambridge, Downing Site, Cambridge, CB2 1QW UK; 2 Present Address: Stem Cell and Brain Research Institute, Université Lyon 1, INSERM U1208, 18 Avenue Doyen Lépine, 69500 Bron, France; 3 Present Address: Laboratoire de Biologie Tissulaire et Ingénierie Thérapeutique, UMS3444 BioSciences Gerland-Lyon Sud, UMR5305, CNRS/Université Lyon 1, Lyon, France; 4 Present Address: CambCol Laboratories Ltd, 18 Oak Lane, Littleport, Ely CB6 1QZ UK

**Keywords:** Cell migration, Cell biology, Cell adhesion, Extracellular matrix, Integrins, Angiogenesis

## Abstract

Blood vessels in the body are lined with endothelial cells which have vital roles in numerous physiological and pathological processes. Collagens are major constituents of the extracellular matrix, and many adherent cells express several collagen-binding adhesion receptors. Here, we study the endothelium–collagen interactions mediated by the collagen-binding integrins, α1β1, α2β1, α10β1 and α11β1 expressed in human umbilical vein endothelial cells (HUVECs). Using qPCR, we found expression of the α10 transcript of the chondrocyte integrin, α10β1, along with the more abundant α2, and low-level expression of α1. The α11 transcript was not detected. Inhibition or siRNA knockdown of the α2-subunit resulted in impaired HUVEC adhesion, spreading and migration on collagen-coated surfaces, whereas inhibition or siRNA knockdown of α1 had no effect on these processes. In tube formation assays, inhibition of either α1 or α2 subunits impaired the network complexity, whereas siRNA knockdown of these integrins had no such effect. Knockdown of α10 had no effect on cell spreading, migration or tube formation in these conditions. Overall, our results indicate that the collagen-binding integrins, α1β1 and α2β1 play a central role in endothelial cell motility and self-organisation.

## Introduction

The vascular endothelium is composed of a monolayer of endothelial cells (ECs) that line the inner surface of blood vessels. The endothelium provides a semi-permeable physical barrier between the solutes in the blood and the surrounding tissues, contributing to the regulation of blood flow and tissue homeostasis^1,2^. The endothelium has also been implicated in the regulation of neutrophil trafficking, hormone trafficking, haemostasis and platelet activation. In pathology, EC dysfunction has been implicated in stroke, heart disease, vascular diseases, diabetes, chronic kidney failure, cancer, atherosclerosis and infectious diseases^1,3,4^. Pathogenic dysregulation of angiogenesis, the growth of new blood vessels from existing vasculature, is associated with wound healing defects, rheumatoid arthritis, diabetic microvascular disease, macular degeneration, ischemia and inflammation^5^. Further, angiogenesis is upregulated in many cancers: for tumours to grow beyond a restricted size they must recruit their own vasculature. Given the array of physiological and pathological processes that involve ECs, it is not surprising that ECs are a major therapeutic target, and to reach this goal understanding the processes that regulate EC behaviour is essential.

The endothelium resides upon an extracellular matrix (ECM) called the basement membrane, which forms a structural surface for organised cell attachment; a process which delivers signals to the cell. Fibrillar and non-fibrillar collagens are major constituents of the blood vessel subendothelial ECM, which also includes fibrinogens, different laminin isoforms, heparan sulphate, entactin and proteoglycans, reviewed by Senger and Davis^5^. Signal transduction between the extracellular matrix and ECs is an important regulator of cell behaviour and ECs themselves modify the composition of the ECM by secreting matrix metalloproteinases (MMPs) or matrix proteins to regulate their turnover^6,7^. ECs adhere to their ECM via receptors such as integrins, a family of 24 transmembrane receptors that form α/β heterodimers from 18 α-subunits and 8 β-subunits. Integrins mediate signal transduction between the ECM and the cytoplasm by linking external signals to the cytoskeleton^8–11^. Integrins play essential roles in embryonic development, cell migration, proliferation, apoptosis, angiogenesis, and haemostasis, and have been implicated in cancer metastasis and inflammation and they are, consequently, promising therapeutic targets^12^.

There are four collagen-binding integrins, α1β1, α2β1, α10β1 and α11β1, with α1β1 and α2β1 also potentially recognising laminin^13,14^. α1β1 and α2β1 are the best-defined in ECs: addition of VEGF to *in-vitro* EC culture upregulates α1β1 and α2β1 expression, cell proliferation, angiogenesis and cell spreading on collagen^15^. Whilst inhibitors of α1β1 and α2β1 impede angiogenesis^16–18^ and inhibiting the synthesis of collagens I and IV, the primary ligands for α1β1 and α2β1, impedes capillary formation^19^, the VEGF-dependent interaction of collagen I with α1β1 and α2β1 induces the activation of the mitogen activated protein kinase (MAPK) pathway, which suppresses apoptosis and promotes cell survival^20,21^. Finally, α1β1 and α2β1 have been implicated in the regulation of both collagen and MMP synthesis^22–24^. Primarily, α10β1 is expressed in chondrocytes^8,10^ and contributes to glioblastoma and sarcoma metastasis^25,26^, migration of melanoma cells^27^ and tumorigenesis. Integrin α11β1 is expressed in fibroblasts, mesenchymal cells, and osteoblasts^28^, and is also a receptor for osteolectin which is involved in osteoblast maturation^29^. Recent work in this laboratory detected α10 at transcript level in freshly prepared HUVECs (Kim, PS-K, PhD Thesis, University of Cambridge, 2014) but whilst α10β1 may be expressed in ECs, little is known about its function in these cells.

Here, the expression and function of the four collagen-binding integrins in HUVECs was explored further. Inhibitors (the small molecule TC-I-15, obtustatin and monoclonal antibody 6F1) and siRNA were used to investigate their role in the regulation of adhesion, cell spreading, migration and tube formation. TC-I-15^30^ is a commercially available broad range inhibitor for α1β1, α2β1 and α11β1^31^. Obtustatin, a 41-residue disintegrin specific for α1β1 found in snake venom, has been used to inhibit angiogenesis and tumour progression in vitro and in vivo^32,33^. 6F1 is a well-characterised inhibitory antibody targeting α2β1^34^. Synthetic triple-helical peptides (THPs)^35^, which contain an active integrin-binding motif flanked by five GPP repeats that maintain triple-helical structure, were used alongside a control peptide, GPP10, consisting of ten GPP repeats without the central active sequence, to model the native collagen structure.

Expression of the mRNA transcript encoding the α10 subunit of α10β1 was detected here in HUVECs alongside small amounts of α1 and an abundance of the α2 transcript. In contrast, α11 was not detected. However, subsequent siRNA knockdown of α10β1 had no effect on the ability of HUVECs to spread or migrate across collagen surfaces or form tubes on 3D Geltrex matrices (a murine Engelbreth-Holm-Swarm tumour derived basement membrane matrix similar to Matrigel). Crucially, an important role for α2β1, and the α2β1 inhibitors 6F1 and TC-I-15, in the regulation of HUVEC migration and tube formation is further characterised, highlighting the importance of collagen-bound α2β1 in endothelial cell biology. Finally, the role of α1β1 is revealed to be more complex, and whilst inhibition of α1β1 with obtustatin did not affect the ability of HUVECs to form tubes or migrate across collagen surfaces, it did diminish the complexity of networks in tube formation assays despite the low expression of α1β1 in these cells.

## Materials and methods

### Materials

Pooled cryopreserved HUVECs were purchased from Promocell, (# C-12208, Lot Numbers and genders: 16Z042 m/m/f/f new-born, 426Z006 m/m/f/m new-born, 426Z007f/f/m/f new-born, 420Z015.2f/m/f/m new-born). Collagen I PureCol® EZ Gel Advanced BioMatrix (# 5074-35ML) and collagen IV (Sigma (# C5533) were used for ECM coatings. TC-I-15 (# 4527/10), Obtustatin (# 4664/100U) and α3β1 (# 2840-A3-050) were purchased from R&D technologies. TC-I-15 was used at 200 µM and obtustatin at 20 µM; 6F1, an α2-inhibitory monoclonal antibody, was the gift of Dr B. Coller, NY, and was used at 10 µg/ml. TC-I-15 is dissolved in 210 µM NaOH to obtain a final molar ratio of 1:1.1 (TC-I-15:NaOH). NaOH alone is used as a vehicle control for TC-I-15 (referred to as TC-I-15 control). Obtustatin and 6F1 are resuspended in PBS. R&D systems anti-α2 MAB12331 and Abcam anti-tubulin ab4074 were used for western blots, along with IRDye® 680 Goat anti-rat and IRDye® 800 anti-rabbit IgG secondary antibodies.

### Cell culture

Pooled HUVECs were cultured in Promocell endothelial growth media 2 (EGM2). Cells were maintained under sterile conditions at 37 °C, 5% CO_2_ without antibiotics. HUVECs were seeded at 500,000 per 75 cm^2^ flask and passaged at 70% confluence using TrypLE^Tm^. HUVECs were used at passage three, four or five. Low passage cells were cryopreserved in Promocell Cryo SFM cell freezing media. All conditions were performed in triplicate and repeated with three different batches of pooled HUVECs, unless stated otherwise in Figure legends.

C2C12 cells, transfected as described previously to express just one of four collagen-binding integrins^36^, were grown in Lifetech DMEM/10% FBS and 1% penicillin/streptomycin, and maintained under sterile conditions at 37 °C, 5% CO_2_. Cells were passaged using Trypsin/EDTA. The C2C12-α10 clone was kindly made available by Dr Evy Lundgren-Åkerlund, Xintela AB, Sweden and the C2C12-α11 clone by Dr Donald Gullberg, U. Bergen Norway.

### Knockdown using siRNA

Two Thermo Silencer Select siRNAs for each target (α1, α2 or α10) were pooled. Each siRNA pool was used at 50 nM combined with the negative control or other siRNA targets to a final siRNA concentration of 150 nM. The Silencer Select siRNAs were, for α1: 4,390,825—s7534, s7532; α2: 4,399,666—s541358, s541359; α10: 4,392,421—s16180, s16181; Negative control #1 4,390,844.

siRNAs were diluted in OptiMEM, 250 μl per well for a 6-well plate. DharmaFECT4 (D4) transfection reagent was diluted in OptiMEM at a 1:50 ratio. 250 μl D4 was then added to the diluted siRNA and incubated for 20 min at RT. Cells were then added in 2 ml EGM2 (without Heparin) per well at 60,000/ml, and incubated for 8 h at 37 °C, 5% CO_2_. The media was then replaced with complete EGM2. siRNA knockdown was validated after 48 h using qRT-PCR. All experiments were carried out 48–72 h post-siRNA transfection.

### Quantitative real-time PCR

The Qiagen RNeasy Plus Mini Kit was used to extract total RNA from HUVECs. cDNA, created using the Taqman reverse transcription reagents, was analysed using the Taqman Gene Expression mastermix in an Applied Biosystems 7300 Real-Time PCR System. TaqMan predesigned primer and probe sets were used and hypoxanthine phosphoribosyltransferase 1 (HPRT1) was used as an endogenous control. (TaqMan HPRT1 Assay 4,448,490, Hs02800695_m1. TaqMan Α1 Assay 4,331,182, Hs00235006_m1. Α2 Assay 4,331,182, Hs00158127_m1. TaqMan Α10 Assay 4,351,372, Hs01006921_m1. TaqMan Α11 Assay 4,331,182, Hs01012939_m1. TaqMan). Relative expression of transcripts was calculated using the ΔCT method. To compare percentage expression relative to the negative control siRNA, ΔΔCT was used. Three technical repeats were carried out with each batch of cDNA.

### Western blots

100,000 cells were lysed directly in Laemmli buffer, lysates were vortexed and boiled for 5 min before loading into NuPage gels using the NuPage Gel Electrophoresis system at 200 V for 45 min. Gels were blotted onto Immobilon-FL PVDF membranes using the BioRad TransBlot® Turbo™ Semi-Dry transfer system. Primary antibodies (rat anti-α2 and rabbit anti-tubulin) were added at 4 °C overnight and the LI-COR secondary antibodies were added for 1 h. Membranes were visualised using a LI-COR Odyssey CLx Western Blot imaging system.

### Cellular static adhesion assays

Immulon 2HB 96-well plates were coated with peptides at 10 μg/ml in 0.01 M acetic acid (or with proteins/antibodies in PBS as stated) overnight at 4 °C. Wells were blocked with sterile filtered 3% BSA in PBS at RT for 1 h. Cells were added at 15,000 or 20,000 cells per well for 1 h in serum-free media. Unbound cells were washed away gently. Remaining cells were lysed with 50 μl per well of 2% Triton X-100 for 1 h at RT. Cell numbers were quantified using the Roche colorimetric cytotoxicity LDH kit, measured as A_490_ in a SpectraMax 190 microplate reader.

### Adhesion assays using xCELLigence™

The real-time xCELLigence RTCA SP electrical impedance system (Acea Biosciences) was used with 96-well RTCA E-plates. Plates were coated with peptides or proteins as before for 1 h at RT or overnight at 4 °C and blocked with 3% BSA in PBS for 1 h at RT. Wells were washed with PBS and equilibrated with 50 μl serum-free media (DMEM or EBM) at 37 °C. A baseline reading was taken before adding 15,000 HUVECs per well in 50 μl of EGM. Readings of Cell Index (CI), a measure of observed impedance of the medium, were taken automatically every 5 min for 4 h. Error bars show SEM calculated from two experimental repeats performed in triplicate.

### Migration random walk assays

Ibidi μ-slide four-well Ph + chamber slides were coated with peptides, proteins or antibodies and blocked as for adhesion assays. 700 μl of HUVECs (14,000/ml) were added in EGM2 and left to attach for 4 h at 37 °C. Then, phase contrast images were taken every 5 min for 9 h using an automated Leica DM6000 microscope (× 10 magnification) in a humidified incubated chamber, 37 °C, 5% CO_2_. ImageJ (TrackMate plugin) was used to quantify migration as average linear distance moved by each cell (Track displacement). Cells visible for over 80 frames were counted; cells that died or left the field of view were not analysed. 10 fields of view from a single well were analysed for each condition in 3 independent repeat experiments using different batches of pooled HUVECs.

### Tube formation assays

Reduced Growth Factor Geltrex (Thermo A1413202) was polymerised in Ibidi angiogenesis μ-slides at 37 °C for 1 h. HUVECs were added (4000 per well) in EGM2 with or without inhibitors or vehicle controls, or 48 h post-siRNA transfection. Phase contrast images were captured at 6 and 24 h and analysed using the *Angiogenesis Analyzer* plugin (G. Carpentier: Angiogenesis Analyzer. ImageJ News, 5 October 2012).

### Cell spreading assays

Cells were seeded on collagen I coated plates either in the presence of inhibitors, or 48 h post-siRNA transfection, for 1 h in EGM2 at 37 °C. Cells were fixed with 4% paraformaldehyde (PFA) in H_2_O and permeabilised with 0.1% Triton X100 in PBS for 5 min. Rhodamine phalloidin and Hoechst 33342 were added for 1 h in PBS with 1% BSA. Cells were imaged using the Leica microscope described above (× 10 magnification). The average cell area (the total area covered by cells divided by the number of stained nuclei) was calculated from 10 fields of view (each 1.005 × 0.754 mm) per well using ImageJ.

### Peptide synthesis and preparation

Collagen-mimetic peptides and derivatives were synthesised as described previously using a CEM Liberty™ microwave-assisted peptide synthesiser^37^. Peptides were cleaved from the resin beads, purified by preparative reverse-phase high performance liquid chromatography, freeze dried and characterized by mass spectrometry. Peptides were dissolved at 5 mg/ml in 0.01 M acetic acid, heated to 70 °C for 5 min and cooled overnight to 4 °C to enable triple helix formation. These were diluted to 10 µg/ml in 0.01 M acetic acid for the coating of empty tissue culture wells prior to experiments.

### Statistical analysis

GraphPad Prism 8.2.1 was used for all statistical tests. Unless otherwise stated in Figure legends, one-way ANOVA, simple or repeated measures, was used throughout, with Dunnett’s multiple comparison test for siRNA experiments and with Sidak’s multiple comparison test to compare inhibitors or activators. The effect of TC-I-15 was compared with its vehicle control, containing NaOH (TC-I-15 control), using t-tests; all other conditions are compared to the medium control. Statistical significance is indicated in Figures or Legends as follows: p < 0.0001****; p < 0.001***; p < 0.01**; p < 0.05*; and ns denotes non-significant. Mean values ± SEM are provided throughout.

## Results

### Integrin α2β1 is highly expressed in HUVECs along with α10β1

**Figure 1:**
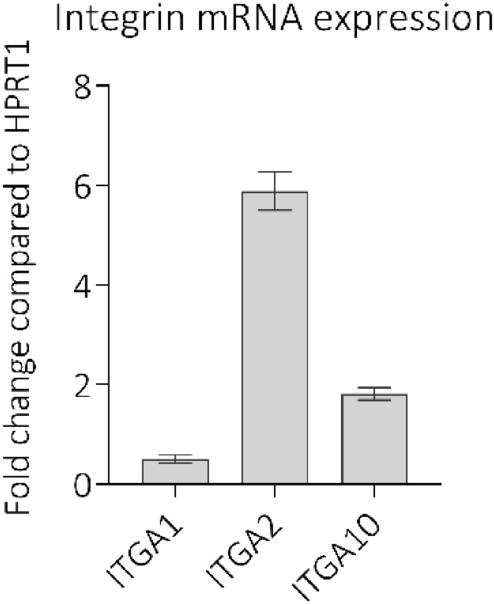
qPCR quantification of relative mRNA expression levels of each integrin, in HUVECs, compared to the endogenous control (HPRT1) using the ∆CT method. ITGA1, ITGA2 and ITGA10 indicate the transcripts that code for the integrin α1, α2 and α10.subunit respectively. For ITGA1, ITGA2 and ITGA10 n = 11. Error bars indicate SEM calculated from all repeats.

The expression of each integrin was probed using TaqMan predesigned primer and probe sets in qPCR assays. HPRT1, previously shown to be stable in endothelial cells^38^, was used as an endogenous control in the ∆CT method. The most abundant integrin in these conditions was α2β1, followed by α10β1 then α1β1 (Fig. 1) and α11β1 was barely detectable (data not shown). Whilst α1β1 and α2β1 have been previously characterised in ECs, mRNA expression of the α10 subunit of α10β1 has only recently been described (Kim, PS-K, unpublished data). The detection of α10β1 suggests a possible novel role for this integrin in endothelial cells, investigated further below.

### TC-I-15 impedes adhesion of HUVECs to collagen mimetic peptide-coated surfaces whereas obtustatin has no effect

**Figure 2:**
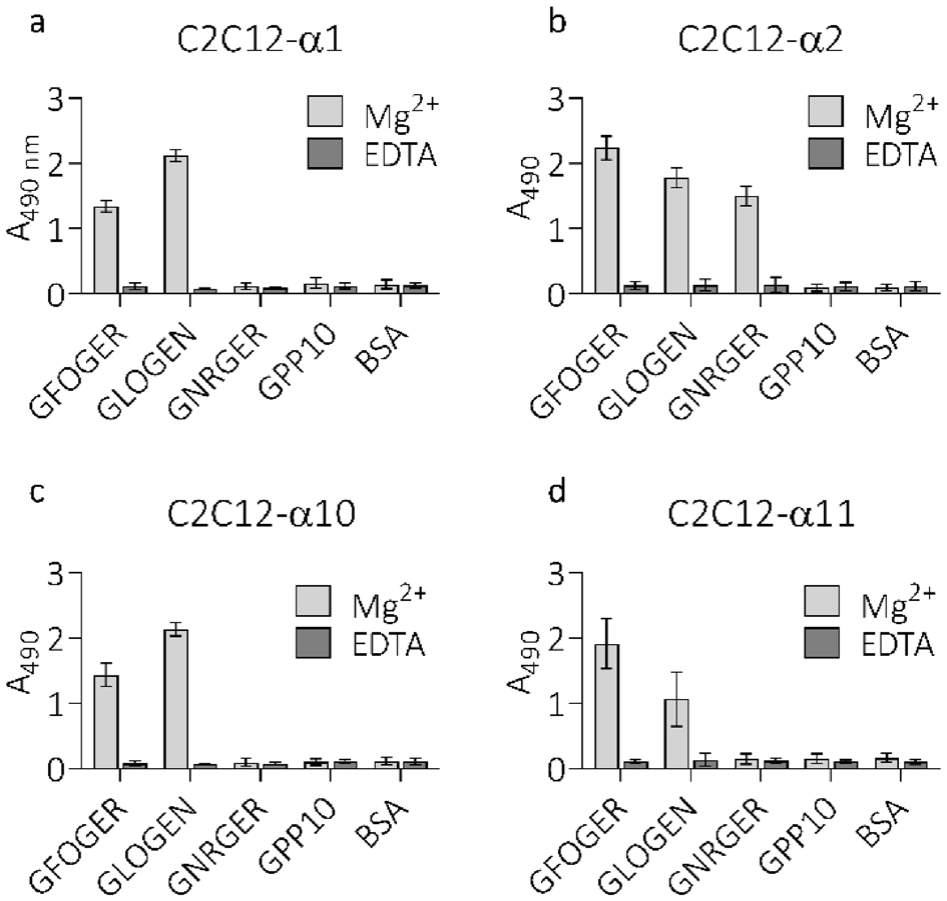
The static adhesion preferences of C2C12 cells that have been stably transfected to express one of the four α1 (**a**), α2 (**b**), α10 (**c**) or α11 (**d**) integrin subunits. Adhesion to collagen-mimetic peptides is shown as A_490_. Light grey bars indicate the presence of 5 mM Mg^2+^ to facilitate integrin adhesion. Dark grey bars indicate the presence of 5 mM EDTA to block integrin binding. Data shown are Mean values ± SEM of three repeats performed in triplicate.

C2C12 cells provide a useful model for investigating the function of the collagen-binding integrins, and were stably transfected with each of the four integrin α subunits (Fig. 2), allowing dimerization with the endogenous mouse β1-subunit to form a functional heterodimer^36^. The integrin binding preferences of these cells were probed by seeding onto collagen-mimetic THPs containing homologues of the integrin-binding motif, GFOGER, which occurs in collagens I, II and in several of the IV α-chains. The four transfected cell lines all adhered strongly to GFOGER, with those expressing α2β1 and α11β1 showing slightly higher affinity. GLOGEN, found in collagen III, also supported strong adhesion of all four lines, with α1β1 and α10β1 transfected cells having slightly higher affinity, whilst GNRGER, occurring in collagens III and XIII, supported adhesion of the α2β1 cell line alone.

**Figure 3:**
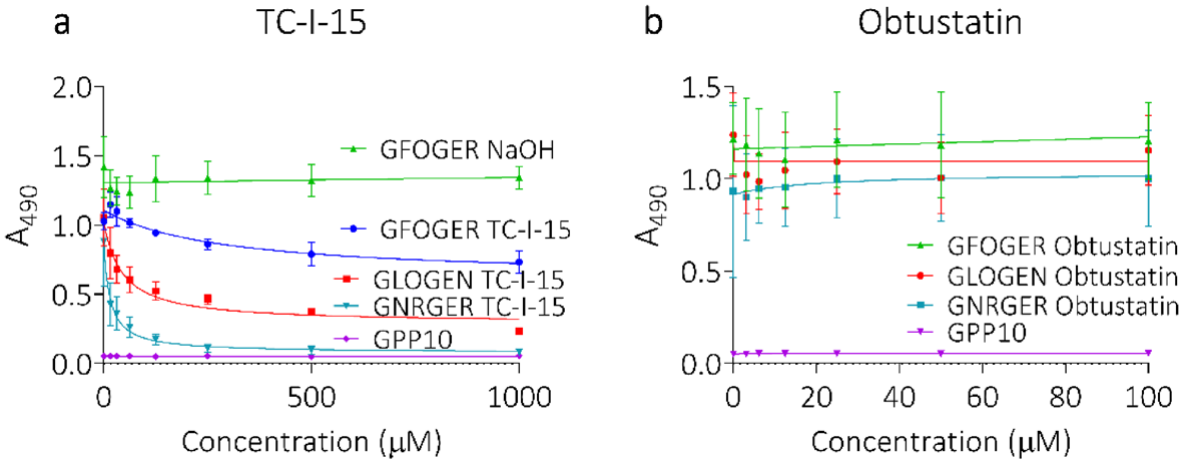
Inhibition of HUVEC adhesion to collagen mimetic peptides using TC-I-15 and Obtustatin. Dose curves for TC-I-15 (**a**) or Obtustatin (**b**) inhibition of HUVEC adhesion to THPs; GFOGER (blue), GLOGEN (red), GNRGER (turquoise) and GPP10 (purple). Cell adhesion is quantified as A_490_. GFOGER NaOH (A—green) shows HUVEC adhesion to GFOGER in the presence of the TC-I-15 vehicle control. For clarity, the vehicle control data was not shown for the other peptides. Error bars indicate SEM calculated from three repeats performed in triplicate.

The inhibitors, TC-I-15 and obtustatin, were tested in HUVECs using the same THPs (Fig. 3a). Obtustatin had no effect on HUVEC adhesion (Fig. 3b), consistent with the specificity of obtustatin for α1β1, recently confirmed^31^, and the low transcript levels of α1β1 observed here in HUVECs. Hence, either α2β1 or α10β1 are sufficient to support full adhesion in the presence of obtustatin. However, TC-I-15, which blocks α1β1, α2β1 and α11β1 (but not α10β1 or α3β1)^31^ inhibited HUVEC adhesion to GFOGER, GLOGEN and GNRGER to varying degrees. Adhesion to GNRGER was abolished completely, consistent with the potent inhibition of α2β1 by TC-I-15 and the specificity of GNRGER for α2β1. The residual adhesion to GFOGER and GLOGEN may reflect α10β1 activity, which is not inhibited by TC-I-15^31^.

### HUVEC adhesion to collagen I, collagen IV and Geltrex is blocked by both TC-I-15 and 6F1

**Figure 4:**
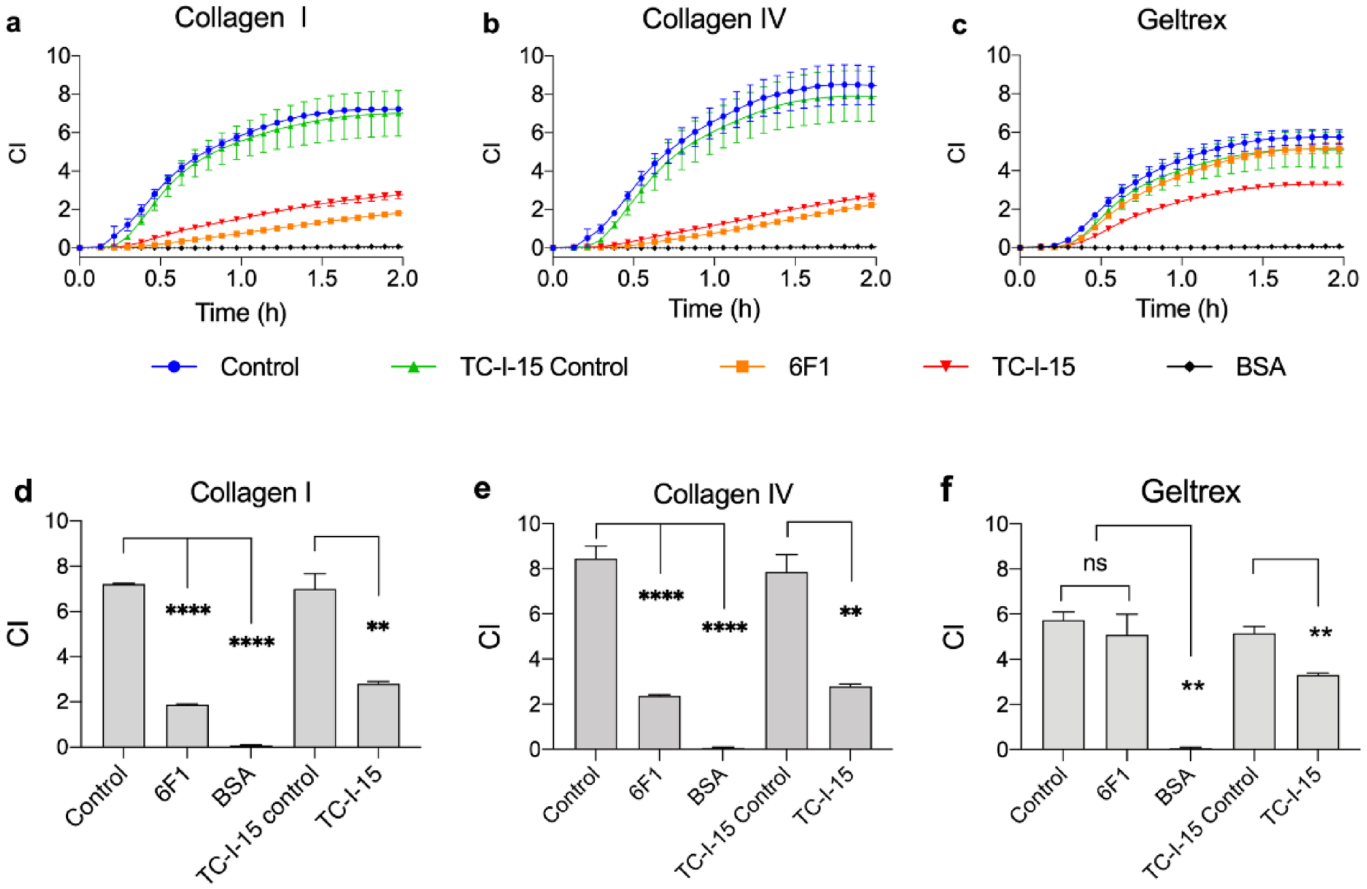
xCELLigence real time adhesion assays determining the effect of integrin inhibition on HUVEC adhesion to (**a**) collagen I, (**b**) collagen IV, and (**c**) Geltrex using 200 µg/ml TC-I-15 (red) and 10 µg/ml 6F1 (orange). Control (blue), TC-I-15 control (green) and BSA (black) are also shown. Panels (**d–f**) show the endpoint analysis of the data. Mean ± SEM from one of two independent repeats performed in triplicate.

The ability of TC-I-15 and 6F1 to impede HUVEC adhesion to collagen I, collagen IV and Geltrex was assessed using the xCELLigence platform (Fig. 4a–c). Adhesion increased with time, reaching a plateau at about 1.5 h under control conditions, and one-way ANOVA of the 2-h timepoint data was used to compare the effects of inhibition (Fig. 4d–f). TC-I-15 and 6F1 each significantly impeded HUVEC adhesion to both collagen I (P = 0.004 and 0.0001, respectively) and collagen IV (P = 0.003 and 0.0001, respectively) compared to their controls. Conversely, 6F1 had no effect on cell adhesion to Geltrex-coated surfaces whilst TC-I-15 treatment resulted in only partial inhibition (P = 0.004).

### Cell adhesion is severely impaired by knockdown of α2, but not of α1 or α10

**Figure 5:**
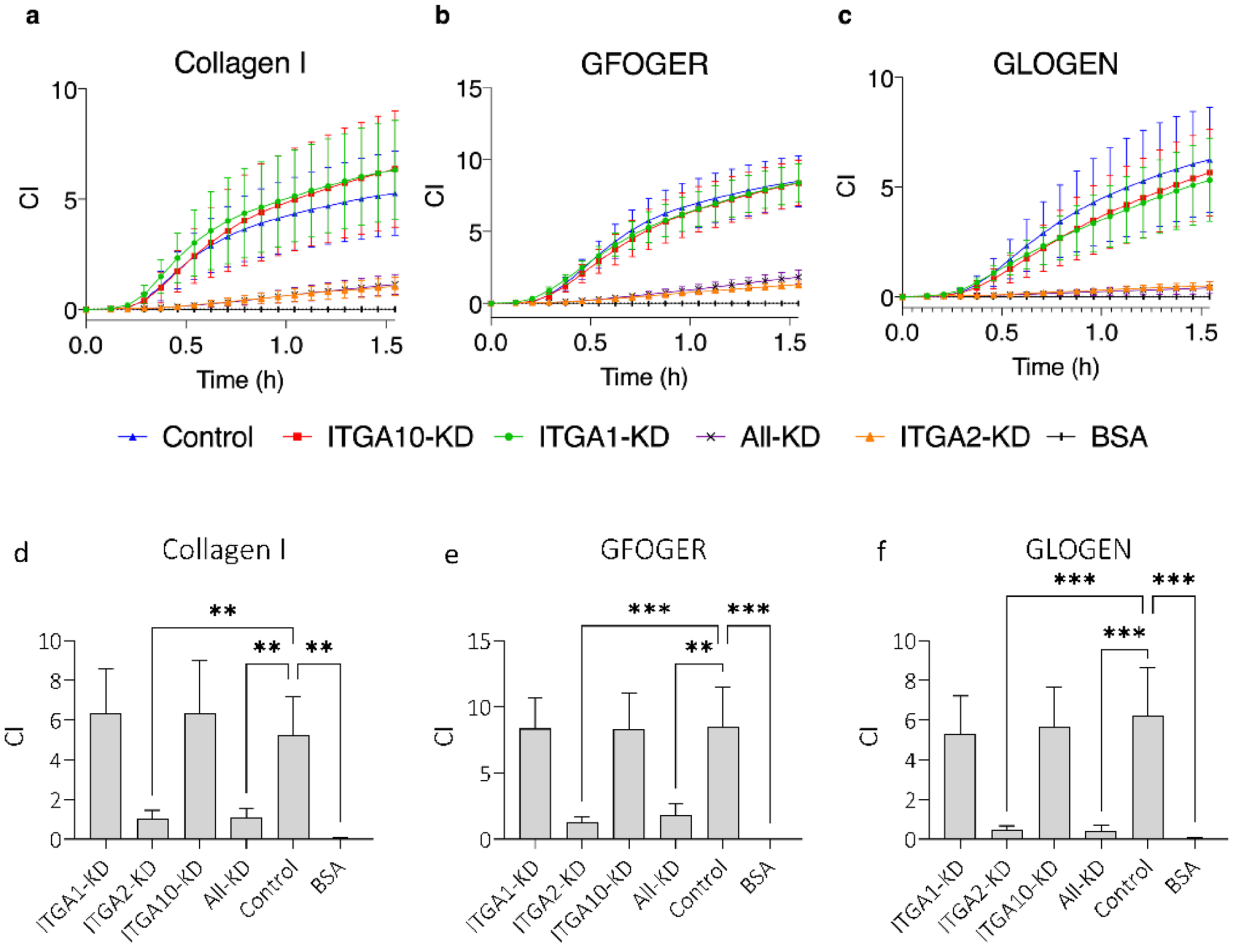
Real-time adhesion of HUVECs to collagen or peptides 48 h post siRNA treatment using xCELLigence. Wells were coated with either (**a**) collagen I, (**b**) GFOGER or (**c**) GLOGEN. The cell index was measured every 5 min. ITGA1-KD (green), ITGA2-KD (orange), ITGA10-KD (red) and All-KD (purple) indicate siRNA knockdown for the transcripts that code for the integrin subunits α1, α2, α10 and the triple knockdown respectively. Control (blue) denotes transfection with the siRNA negative control. BSA (black) shows cell attachment to BSA blocked wells as a baseline. Corresponding mean endpoint adhesion data is shown in (**d–f**) and statistical significance was determined using two-way ANOVA. Error bars in all panels indicate SEM calculated from three repeats performed in triplicate.

Using the xCELLigence system, HUVEC attachment to collagen I, GFOGER and GLOGEN was investigated after siRNA knockdown of α1, α2, α10, all three subunits together, or treatment with an siRNA negative control (Fig. 5). An 85–90% reduction of mRNA expression was observed 48 h post-siRNA treatment, and this translated to a 90% reduction at the protein level in western blots (Supplemental Information: Fig. S1). Knockdown of α2, or the triple knockdown of all three α subunits, severely diminished cell attachment to collagen I, GFOGER and GLOGEN, shown by a dramatic decrease in cell index compared to the control. Conversely, siRNA knockdown of α1 or α10 had no effect on cell attachment to collagen I, GFOGER or GLOGEN. In all three cases, siRNA knockdown of α2 alone produces very similar results to the triple knockdown of all three subunits, and adhesion is reduced to almost baseline levels. This suggests that HUVECs use α2β1 as their main collagen-binding adhesion receptor.

### TC-I-15 and 6F1 impede cell spreading along with siRNA knockdown of α2β1 or all three integrins

**Figure 6:**
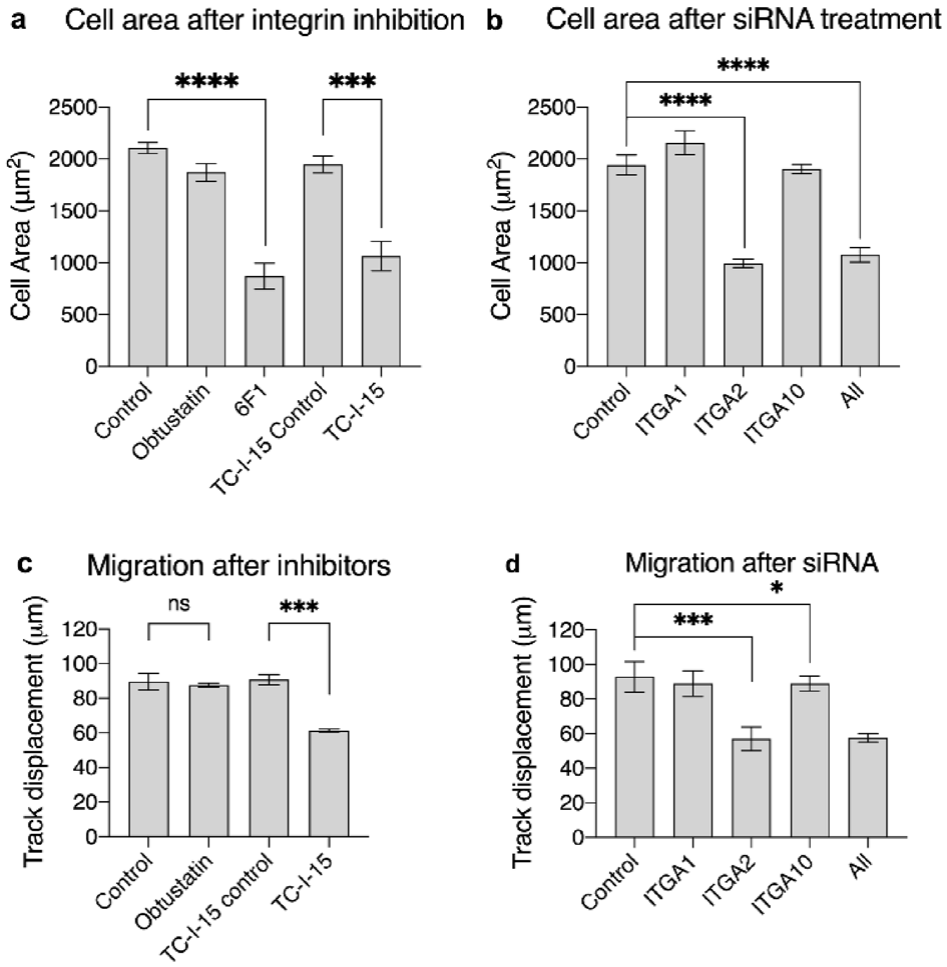
(**a,b**) Quantification of fluorescence microscopy images of HUVEC cell spreading on collagen I coated surfaces (**a**) in the presence or absence of inhibitors or vehicle controls or (**b**) 48 h post siRNA knockdown of the indicated integrin α subunits. The quantification of cell spreading was determined by measuring the average cell area on 10 randomly selected fields of view using ImageJ. (**c,d**) Quantification of HUVEC migration after integrin inhibition or siRNA treatment, shown as distance migrated. Cells were seeded onto collagen I coated surfaces in either (**a**) the presence of absence of inhibitors or vehicle controls or (**b**) 48 h after siRNA treatment. The ImageJ plugin Trackmate was used to track and quantify the movement of each cell. 10 fields of view were analysed for each condition, for three repeats. Error bars indicate SEM.

We examined the effect of integrin inhibitors on HUVEC spreading after adhesion to surfaces coated with collagen I (Fig. 6a). Obtustatin had no effect, consistent with the cell adhesion experiments (Fig. 3) where α2β1 and/or α10β1 are the main collagen receptors supporting cell spreading. TC-I-15 inhibition of α2β1 and α1β1 severely impeded HUVEC cell spreading on collagen I- and IV-coated surfaces (Figs. 6a and S4) compared to the control, despite the presence of any uninhibited α10β1 activity. The sole inhibition of α2β1 using 6F1 significantly reduced mean cell area on both collagen I (P < 0.0001) and IV (P < 0.001). Further, the dual inhibition of α2β1 and α1β1 with TC-I-15 did not appear to impede HUVEC spreading more than inhibition of α2β1 with 6F1 alone, suggesting that α1β1 plays at best a minor role in cell spreading. This is again consistent with the low mRNA expression of the α1 subunit shown in Fig. 1.

Figure 6b shows HUVEC cell spreading over collagen I coated surfaces, 48 h after siRNA knockdown of integrins or siRNA negative control treatment. Here, siRNA knockdown of α1 or α10 had no effect on HUVEC cell spreading compared to the control, presumably because α2β1 expression predominates. Knockdown of α2, however, severely impeded cell spreading (P < 0.0001) identical to the effect of the triple knockdown (P < 0.0001).

### HUVEC migration over collagen I-coated surfaces is impeded by inhibition or siRNA knockdown of α2β1

Random walk migration of HUVECs was measured in the presence or absence of inhibitors, or 48 h after siRNA knockdown (Fig. 6c,d). Figure 6a shows that dual inhibition of α1β1 and α2β1 using TC-I-15 decreased the distance migrated compared to the vehicle control (P = 0.0002), whereas inhibition of α1β1 alone using obtustatin had no effect. Further, siRNA knockdown of α1 or α10 had no effect on the distance migrated compared to the negative control, but knockdown of α2 or the triple knockdown significantly reduced the distance migrated (Fig. 6b).

### Angiogenesis is inhibited by TC-I-15 and 6F1

**Figure 7:**
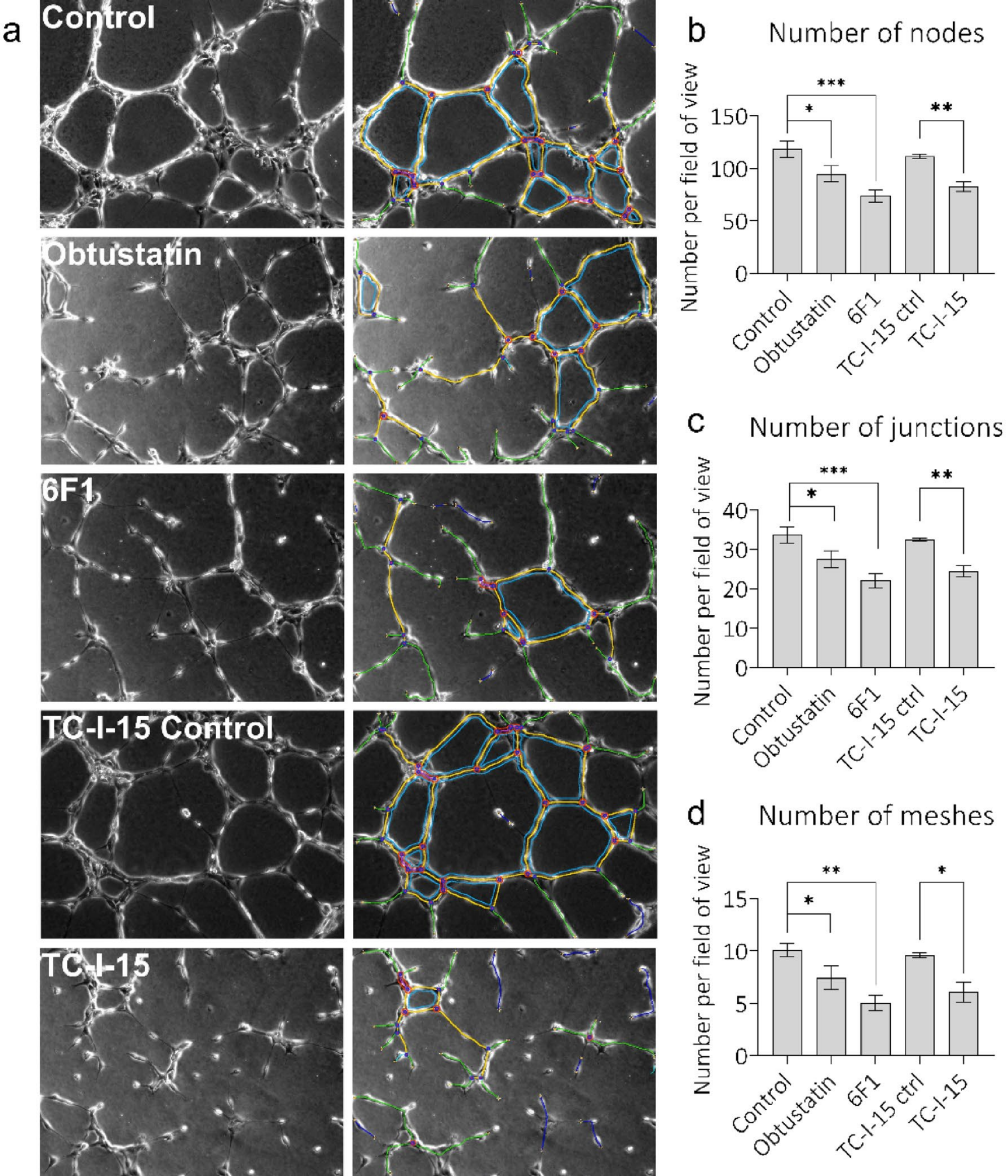
Quantification of tube formation after integrin inhibition. (**a**) Phase images taken 6 h after seeding cells in the presence or absence of inhibitors or vehicle controls. The left column of images indicates phase contrast images, the right column shows the angiogenesis analyser overlay. The quantification of (**b**) nodes, (**c**) junctions and (**d**) meshes. Error bars indicate SEM calculated from three repeats performed in triplicate.

**Figure 8:**
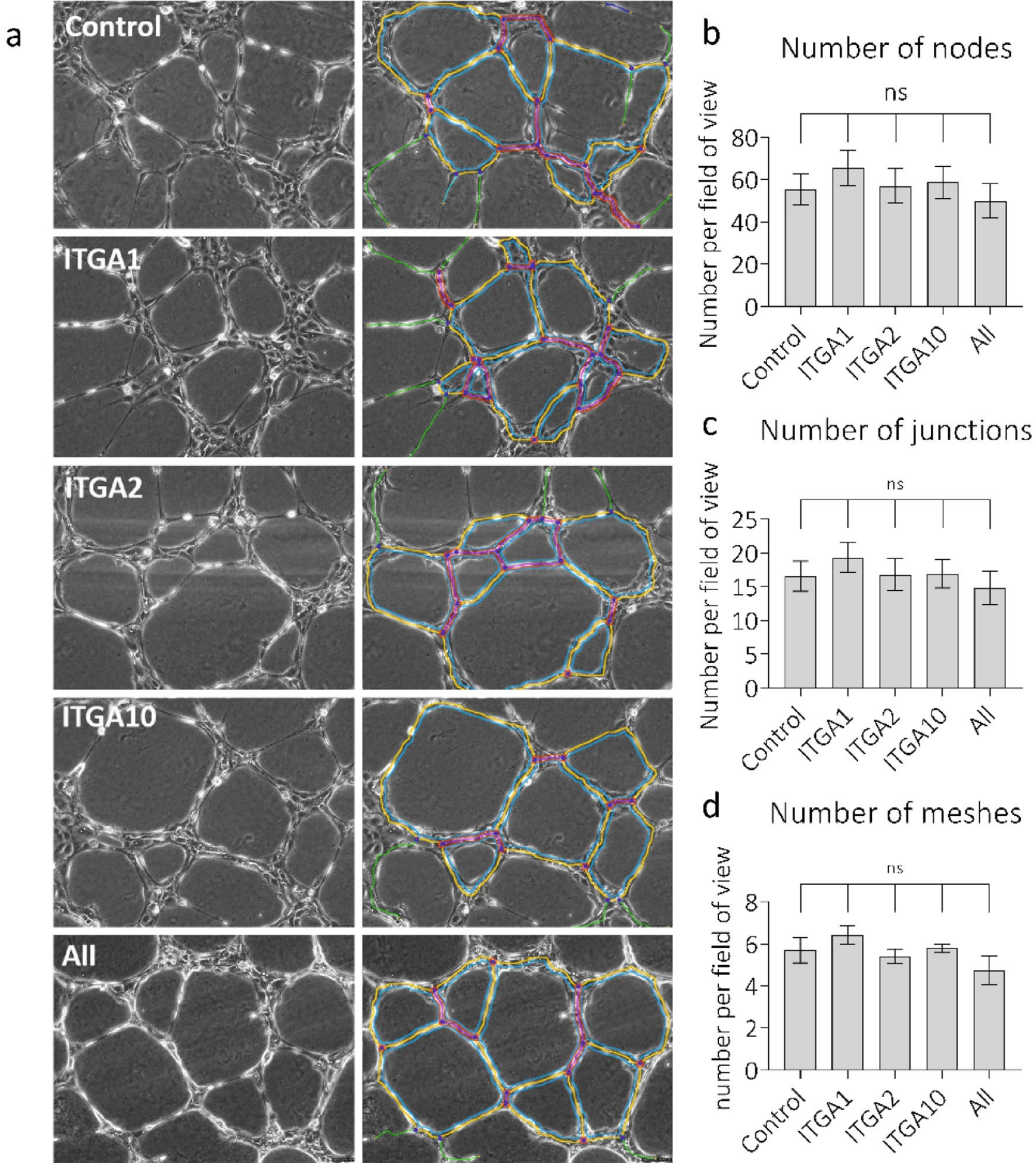
Quantification of tube formation after siRNA treatment. (**a**) Phase contrast images taken 6 h after seeding siRNA treated HUVECs on Geltrex 3D matrices. HUVECs were seeded 48 h after siRNA treatment. The left column of images indicates phase contrast images, the right column indicates the angiogenesis analyser overlay. The quantification of tube formation as the number of (**b**) nodes, (**c**) junctions or (**d**) meshes per field of view after knockdown of the indicated integrin α subunits. Error bars indicate SEM calculated from three repeats performed in triplicate.

To probe the roles of these integrins in a more complex system, tube formation assays using 3D gels made from Geltrex, were carried out in the presence or absence of inhibitors, and 48 h after siRNA treatment (Fig. 7). Nodes, Junctions and Meshes were the three parameters chosen for quantification. The Nodes function quantifies points where branches of the network converge, a Junction is a collection of nodes, and a Mesh is an area entirely closed off by surrounding branches^39^. All three parameters are, therefore, measurements of HUVEC network complexity. Inspection of the images suggests incomplete network formation in the presence of each inhibitor, which is borne out by the analysis. Firstly, the single inhibition of α1β1 by Obtustatin significantly decreased the number of Nodes (P = 0.008), Junctions (P = 0.009) and Meshes (P = 0.038). Moreover, the single inhibition of α2β1 using 6F1 also decreased the number of Nodes (P = 0.0003), Junctions (P = 0.0005) and Meshes (P = 0.0012) but to a further extent than Obtustatin, coherent with the more abundant expression of α2β1. Finally, dual inhibition of α1β1 and α2β1 using TC-I-15, significantly decreased the number of Nodes (P = 0.011), Junctions (P = 0.0115) and Meshes (P = 0.0195) per field, in a manner comparable to 6F1. In marked contrast, after siRNA knockdown there were no significant changes in the number of nodes, junctions or meshes for any condition compared to the negative siRNA control (Fig. 8).

## Discussion

The work presented here describes novel expression of the chondrocyte-associated integrin, α10β1, in HUVECs at the mRNA transcript level. Here, the most abundant transcript was α2, followed by α10 and then α1, whilst α11 was not detected. As α1β1 and α2β1 but not α10β1 have been characterised in endothelial cells previously, it was important to investigate the roles of α10β1 in ECs. Therefore, inhibitors and siRNA knockdowns were used to probe the functions of these three collagen-binding integrins expressed in ECs. Using siRNA, an 85–90% knockdown was observed at the mRNA and protein levels (Fig. S1) but there was no compensation between the subunits after single siRNA knockdown of each subunit. For example, when α2 mRNA is depleted, there was no increase in α1 or α10 mRNA to compensate for the loss of α2.

Inhibition of the integrins investigated here would be expected to disrupt HUVEC adhesion to collagen. Integrin-mediated adhesion stabilises the actin cytoskeletal-ECM interaction at membrane protrusions leading to decreased actin retrograde flow and increased protrusion in filopodia and lamellipodia, contributing to cell spreading^40^, and so these would also be expected to be modulated by perturbation of expression or function. When integrins adhere to the ECM they cluster, together and with other adhesion receptors, to form adhesion complexes, such as nascent adhesions that can mature into focal adhesions^40–42^. Integrin-mediated adhesion at the leading-edge of a migrating cell will facilitate traction with the ECM which pulls the rest of the cell forward, thus also promoting cell migration. Here, inhibition of α2β1 using the monoclonal antibody 6F1 or inhibition of α1β1 and α2β1 using the small molecule TC-I-15 each resulted in impaired adhesion and/or cell spreading on collagen I, collagen IV (Fig. S4), and related collagen-mimetic peptides in both static and real-time adhesion and cell spreading assays. In adhesion assays, the level of inhibition using TC-I-15 was dependent on the affinity of the peptide coating, and higher concentrations of TC-I-15 were needed to impede adhesion to GFOGER than to GLOGEN, in line with a competitive mode of inhibition^30,31^. TC-I-15 and 6F1 also markedly reduced cell spreading on collagen I with HUVECs displaying irregular cell morphology (Fig. S2), likely due to the disrupted formation of adhesion complexes in filopodia and lamellipodia in the absence of functional α2β1. Conversely, only dual inhibition of both α1β1 and α2β1 using TC-I-15 was sufficient to impede HUVEC adhesion to Geltrex, a more complex ECM substitute. The residual adhesion and spreading on collagen surfaces and THPs seen in the presence of TC-I-15 and 6F1 might be attributed to the presence of the still active α10β1, although we are not aware of specific inhibitors of α10β1 to test this directly. The results here highlight the importance of α2β1 in facilitating HUVEC cell adhesion and spreading on collagen, THPs and Geltrex. In contrast, obtustatin inhibition of α1β1 had no effect on HUVEC cell adhesion, spreading or migration, likely due to the low level of α1β1 expression and the presence of the more abundant integrin, α2β1, despite the presence of collagen IV in Geltrex, for which α1β1 is a preferential ligand.

Similarly, HUVEC adhesion to collagen, GFOGER and GLOGEN was also affected by siRNA knockdown of α2β1 or all three integrins which resulted in a striking decrease in cell index in xCELLigence real-time adhesion assays, reduced to near-baseline levels, using collagen I, GFOGER and especially GLOGEN. No inhibition of adhesion was seen in the siRNA knockdowns of α1β1 or α10β1, because α2β1 is likely sufficient to override the effects of depleting the much less abundant α1β1 or α10β1. The loss of adhesion in the single α2β1 knockdown was remarkably similar in amplitude to the triple knockdown in all conditions, using collagen-coated surfaces and integrin-specific peptides, again suggesting that HUVECs use α2β1 as their main collagen-binding integrin. This inhibition of adhesion was also seen in cell spreading assays where cells failed to spread and displayed irregular morphology after siRNA knockdown of α2β1 or the triple knockdown (Fig. S3).

The impaired adhesion seen in the presence of TC-I-15 and 6F1 and in the siRNA knockdown of α2β1 or all three integrins translated to a decrease in cell migration across collagen-coated surfaces. Again, this may be partly attributed to the disruption of adhesion complexes in filopodia and lamellipodia in the absence of α2β1. The effect of specific inhibition or knockdown of α2β1 in these assays was similar in extent to that of the dual inhibitor or triple knockdown, suggesting that α1β1 and α10β1 are not crucial for these various processes. Inhibition or knockdown of α2β1 alone is enough to attain maximal inhibition of migration. In contrast, inhibition or siRNA knockdown of α1β1 alone had no effect on HUVEC migration across collagen surfaces, most likely due to presence of the more abundant α2β1 and α10β1. However, during migration assays there were no differences in cell morphology across conditions and the cells were able to attach, spread and migrate in all conditions, albeit more slowly. The migration assays take place over a much longer time and, eventually, the cells find a way to attach to the collagen coated surfaces in the absence of these integrins, either through other collagen receptors or through synthesis and secretion of extracellular matrix proteins and their associated receptors. Although beyond the scope of this study, it would be interesting to see if HUVECs upregulate the expression of other receptors or matrix proteins in the absence of the collagen-binding integrins.

In tube formation assays the decrease in adhesion, spreading and migration in the presence of TC-I-15 and 6F1 translated into decreased network complexity in all parameters measured. Furthermore, an additional modest decrease in the network complexity was also observed in the presence of obtustatin, suggesting that despite the low levels of α1β1 expression, there is a role for this integrin in the more complex Geltrex tube formation system. In previous assays, collagen I or peptide coatings were used in simplified systems, however for tube formation assays using Geltrex, the presence of other matrix proteins such as laminin, elastin and proteoglycans, may alter α1β1 expression or function. This could explain the decrease in network complexity caused by obtustatin mediated inhibition of α1β1, whereas no such decrease was observed for adhesion, cell spreading or migration assays using specific collagenous substrates.

There is conflicting evidence for the role of integrin α2β1 in regulating angiogenesis. Both knockdowns and inhibitors have been used in mice to investigate this question and the resulting data reflects a complicated regulatory landscape. For example, one study found that α2β1 knockout mice exhibited increased tumour angiogenesis when challenged with PLGF expressing cancer cells, suggesting an anti-angiogenic role for α2β1^43^. A second α2β1 knockout study also found that α2β1 deficient mice showed increased angiogenesis in wound healing experiments at 10 days post-injury, and that sponges implanted in α2β1-null mice showed increased angiogenesis^44^. In contrast, a third study found that α2β1-null mice showed no differences in wound healing or angiogenesis^45^.

On the other hand, inhibition of α2β1 results in a different phenotype. A study in which mice were injected with Matrigel containing hVEGF_165_-transfected cells found that inhibitory antibodies targeting α1β1 or α2β1 suppressed VEGF-stimulated angiogenesis in the dermis. In the same study, inhibition of α1β1 and α2β1 simultaneously suppressed tumour angiogenesis after A431 carcinoma cells were implanted into mice^20^, suggesting that both α1β1 and α2β1 are pro-angiogenic. Another study also found that blocking α1β1 and α2β1 using inhibitory antibodies resulted in reduced formation of new, small blood vessels without affecting existing vasculature^16^. Finally, endorepellin, an anti-angiogenic fragment of perlecan, requires both α2β1 and VEGF receptors to enact its anti-angiogenic effects^46^ and endorepellin treatment results in its selective localisation to tumour vasculature resulting in decreased tumour angiogenesis in mice^47^. A host of α1β1 inhibitors such as obtustatin and arresten have also been shown to reduce angiogenesis^32,33,48,49^. Additionally, in α1β1-null mice, implanted tumours show reduced tumour vascularisation due to increased MMP7- and MMP9-mediated production of angiostatin^50^. Here, while the inhibition of α2β1, and to a lesser extent α1β1, disturbs tube formation, siRNA knockdown of any, or all, of these integrins has no effect on HUVEC ability to form networks of tubes on 3D Geltrex gels. The discrepancies seen here, and in the literature, between inhibition of integrins and knockout or knockdown of a receptor is likely due in part to the inherent differences between inhibition and knockdown. As inhibitors work quickly, the cell must respond rapidly to the inhibition of integrins in each setting. In contrast, with siRNA, the cells have 48 h in which to register the decrease in integrin signalling and compensate for this loss. This compensation could either be upregulation of other collagen-binding adhesion proteins or, in the case of tube formation, upregulation of other integrins or matrix binding proteins. As an example, laminin is a major constituent of Geltrex, so HUVECs could upregulate the expression of laminin receptors such as α3β1 and α5β1. Also, in the presence of inhibitors, the cell might receive an active non-ligated signal from the inhibited integrin which conveys its non-adherent state. In contrast, in the absence of the receptor after siRNA knockdown or genetic ablation, this hypothetical non-ligated signal could not occur. This could affect downstream signalling pathways involved in tube formation. Furthermore, the mode of inhibition is important when considering the downstream signalling events. A competitive ligand that mimics collagen could result in integrin activation and positive downstream signalling, whereas an inhibitor like TC-I-15, which stabilises the inactive conformation will result in inactive downstream signalling. Finally, integrins form focal adhesions, which contain many other signalling molecules and scaffolding proteins, in the absence of integrin expression these focal adhesions will have a different composition that could affect the downstream signalling processes. For example, α3β1 has been found in focal adhesions even when its ligand is not present^51^. In our case, inhibition of α2β1 disrupts tube formation because HUVECs rely on this integrin to facilitate appropriate adhesion to the ECM, when we knockdown this integrin the cells have time to upregulate expression of other ECM receptors to compensate for the loss of α2β1 and there is therefore no disruption of tube formation.

Integrins exist in active/open or inactive/closed conformations. In the inactive/closed conformation, the C-helix blocks adhesion of the ligand to the αI-domain MIDAS. In the active/open conformation, helix 7 moves downward to coordinate the Mg^2+^ ion in the βI-domain and the resulting conformational changes increase ligand affinity drastically^52,53^. TC-I-15 is thought to interact with the αI/βI-domain interface to stop movement of the helix-7 in the αI-domain, thus stabilising the inactive conformation^30^. TC-I-15 has been previously characterised in our hands as a broad inhibitor for α1β1, α2β1 and α11β1, with no inhibition seen for α10β1, and no effect on adhesion of another non-I-domain-containing integrin, α3β1^31^.

Since α1β1 and α2β1 have been implicated in the regulation of proliferation and angiogenesis it is not surprising that they are both also implicated in cancer progression. α1β1 is upregulated in colorectal cancer^54^ and its knockdown or inhibition results in reduced tumour progression^55^ and decreased tumour angiogenesis^32^. Both α1β1 and α2β1 enhance cancer cell migration and metastasis, for example, by upregulating MMP synthesis via MAPK signalling^56^. α2β1 also promotes prostate cancer metastasis to the skeleton, resulting in a poor prognosis for patients^57^. In our hands, inhibition or knockdown of α1β1, α2β1 or α10β1 had no effect on proliferation (Supporting Information, Fig. S5). However, different EC subtypes have specific phenotypes, and, in this study, we focused solely on HUVECs whereas other publications involved more specialised EC subtypes, for example microvascular ECs, and this could explain the lack of effect on proliferation seen here. Nevertheless, the inhibition of angiogenesis and/or migration in the presence of 6F1, obtustatin and TC-I-15 here presents a possible avenue for disrupting tumour angiogenesis.

With respect to cardiovascular research, the work presented here contributes to the fundamental understanding of the interactions between ECs and their surrounding ECM. Our data highlights the importance of collagen-binding integrins in the regulation of EC behaviour. While α10β1 was found to have no visible effect on adhesion, spreading, migration or tube formation, the only suggestion of a role for α1β1 observed here was in angiogenesis. It would be interesting to discover whether, after seeding HUVECs into the complex Geltrex milieu, this resulted from an increase in α1β1 expression or from a specific function related to angiogenesis. In contrast, α2β1 was shown to be crucial to HUVEC’s affinity for collagen-containing or collagen-mimicking substrates. Our results demonstrate that, as a consequence, α2β1 is essential for HUVEC motility and network organization.

## Supplementary Information

Supplementary Figures.

## Data Availability

The datasets generated during and/or analysed during the current study are not publicly available due to limited online storage capabilities but are available from the corresponding author on reasonable request.

## References

[1] Rajendran P (2013). The vascular endothelium and human diseases. Int. J. Biol. Sci..

[2] Alberts B (2002). Molecular Biology of the Cell.

[3] Roberts AC, Porter KE (2013). Cellular and molecular mechanisms of endothelial dysfunction in diabetes. Diabetes Vasc. Dis. Res..

[4] Cines DB (1998). Endothelial cells in physiology and in the pathophysiology of vascular disorders. Blood.

[5] Senger DR, Davis GE (2011). Angiogenesis. Cold Spring Harb. Perspect. Biol..

[6] Neve A (2014). Extracellular matrix modulates angiogenesis in physiological and pathological conditions. Biomed. Res. Int..

[7] Pozzi A, Zent R (2009). Regulation of endothelial cell functions by basement membrane- and arachidonic acid-derived products. Wiley Interdiscip. Rev. Syst. Biol. Med..

[8] Barczyk M, Carracedo S, Gullberg D (2010). Integrins. Cell Tissue Res..

[9] Gahmberg CG (2009). Regulation of integrin activity and signalling. Biochim. Biophys. Acta.

[10] Takada Y, Ye X, Simon S (2007). The integrins. Genome Biol..

[11] Arnaout MA, Mahalingam B, Xiong JP (2005). Integrin structure, allostery, and bidirectional signaling. Annu. Rev. Cell Dev. Biol..

[12] Alique M (2014). Integrin-linked kinase plays a key role in the regulation of angiotensin II-induced renal inflammation. Clin. Sci. (Lond.).

[13] Hall DE (1990). The alpha 1/beta 1 and alpha 6/beta 1 integrin heterodimers mediate cell attachment to distinct sites on laminin. J. Cell Biol..

[14] Elices MJ, Hemler ME (1989). The human integrin VLA-2 is a collagen receptor on some cells and a collagen/laminin receptor on others. Proc. Natl. Acad. Sci. U.S.A..

[15] Garmy-Susini B, Varner JA (2008). Roles of integrins in tumor angiogenesis and lymphangiogenesis. Lymphat. Res. Biol..

[16] Senger DR (1997). Angiogenesis promoted by vascular endothelial growth factor: Regulation through alpha1beta1 and alpha2beta1 integrins. Proc. Natl. Acad. Sci. U.S.A..

[17] San Antonio JD (2009). A key role for the integrin alpha2beta1 in experimental and developmental angiogenesis. Am. J. Pathol..

[18] Niland S, Eble JA (2012). Integrin-mediated cell-matrix interaction in physiological and pathological blood vessel formation. J. Oncol..

[19] Haralabopoulos GC (1994). Inhibitors of basement membrane collagen synthesis prevent endothelial cell alignment in matrigel in vitro and angiogenesis in vivo. Lab. Investig..

[20] Senger DR (2002). The alpha(1)beta(1) and alpha(2)beta(1) integrins provide critical support for vascular endothelial growth factor signaling, endothelial cell migration, and tumor angiogenesis. Am. J. Pathol..

[21] Byzova TV (2000). A mechanism for modulation of cellular responses to VEGF: Activation of the integrins. Mol. Cell.

[22] Riikonen T (1995). Integrin alpha 2 beta 1 is a positive regulator of collagenase (MMP-1) and collagen alpha 1(I) gene expression. J. Biol. Chem..

[23] Gardner H (1999). Absence of integrin alpha1beta1 in the mouse causes loss of feedback regulation of collagen synthesis in normal and wounded dermis. J. Cell Sci..

[24] Ronziere MC (2005). Integrin alpha1beta1 mediates collagen induction of MMP-13 expression in MC615 chondrocytes. Biochim. Biophys. Acta.

[25] Munksgaard Thoren M (2019). Integrin alpha10, a novel therapeutic target in glioblastoma, regulates cell migration, proliferation, and survival. Cancers (Basel).

[26] Okada T, Singer S (2017). Integrin-alpha10 drives tumorigenesis in sarcoma. Oncoscience.

[27] Wenke AK (2007). Expression of integrin alpha10 is induced in malignant melanoma. Cell Oncol..

[28] Zeltz C, Gullberg D (2016). The integrin-collagen connection—A glue for tissue repair?. J. Cell Sci..

[29] Shen B (2019). Integrin alpha11 is an Osteolectin receptor and is required for the maintenance of adult skeletal bone mass. Elife.

[30] Miller MW (2009). Small-molecule inhibitors of integrin alpha2beta1 that prevent pathological thrombus formation via an allosteric mechanism. Proc. Natl. Acad. Sci. U.S.A..

[31] Hunter EJ (2021). Selectivity of the collagen-binding integrin inhibitors, TC-I-15 and obtustatin. Toxicol. Appl. Pharmacol..

[32] Marcinkiewicz C (2003). Obtustatin: A potent selective inhibitor of alpha1beta1 integrin in vitro and angiogenesis in vivo. Cancer Res..

[33] Brown MC (2008). Angiostatic activity of obtustatin as alpha1beta1 integrin inhibitor in experimental melanoma growth. Int. J. Cancer.

[34] Coller BS (1989). Collagen-platelet interactions: Evidence for a direct interaction of collagen with platelet GPIa/IIa and an indirect interaction with platelet GPIIb/IIIa mediated by adhesive proteins. Blood.

[35] Farndale RW (2008). Cell-collagen interactions: The use of peptide toolkits to investigate collagen-receptor interactions. Biochem. Soc. Trans..

[36] Tiger CF (2001). Alpha11beta1 integrin is a receptor for interstitial collagens involved in cell migration and collagen reorganization on mesenchymal nonmuscle cells. Dev. Biol..

[37] Malcor JD (2016). The synthesis and coupling of photoreactive collagen-based peptides to restore integrin reactivity to an inert substrate, chemically-crosslinked collagen. Biomaterials.

[38] Wei R, Stewart EA, Amoaku WM (2013). Suitability of endogenous reference genes for gene expression studies with human intraocular endothelial cells. BMC Res. Notes.

[39] Carpentier G (2020). Angiogenesis Analyzer for ImageJ—A comparative morphometric analysis of "endothelial tube formation assay" and "fibrin bead assay". Sci. Rep..

[40] Huttenlocher A, Horwitz AR (2011). Integrins in cell migration. Cold Spring Harb. Perspect. Biol..

[41] Guo WH, Wang YL (2007). Retrograde fluxes of focal adhesion proteins in response to cell migration and mechanical signals. Mol. Biol. Cell.

[42] Ridley AJ (2003). Cell migration: Integrating signals from front to back. Science.

[43] Zhang Z (2008). Alpha2beta1 integrin expression in the tumor microenvironment enhances tumor angiogenesis in a tumor cell-specific manner. Blood.

[44] Zweers MC (2007). Integrin alpha2beta1 is required for regulation of murine wound angiogenesis but is dispensable for reepithelialization. J. Investig. Dermatol..

[45] Chen J (2002). The alpha(2) integrin subunit-deficient mouse: A multifaceted phenotype including defects of branching morphogenesis and hemostasis. Am. J. Pathol..

[46] Goyal A (2011). Endorepellin, the angiostatic module of perlecan, interacts with both the alpha2beta1 integrin and vascular endothelial growth factor receptor 2 (VEGFR2): A dual receptor antagonism. J. Biol. Chem..

[47] Bix G (2006). Endorepellin in vivo: Targeting the tumor vasculature and retarding cancer growth and metabolism. J Natl. Cancer Inst..

[48] Nyberg P (2008). Characterization of the anti-angiogenic properties of arresten, an alpha1beta1 integrin-dependent collagen-derived tumor suppressor. Exp. Cell Res..

[49] Boosani CS (2010). Inhibitory effects of arresten on bFGF-induced proliferation, migration, and matrix metalloproteinase-2 activation in mouse retinal endothelial cells. Curr. Eye Res..

[50] Pozzi A (2000). Elevated matrix metalloprotease and angiostatin levels in integrin alpha 1 knockout mice cause reduced tumor vascularization. Proc. Natl. Acad. Sci. U.S.A..

[51] DiPersio CM, Shah S, Hynes RO (1995). Alpha 3A beta 1 integrin localizes to focal contacts in response to diverse extracellular matrix proteins. J. Cell Sci..

[52] Emsley J (1997). Crystal structure of the I domain from integrin alpha2beta1. J. Biol. Chem..

[53] Emsley J (2000). Structural basis of collagen recognition by integrin alpha2beta1. Cell.

[54] Boudjadi S, Carrier JC, Beaulieu JF (2013). Integrin alpha1 subunit is up-regulated in colorectal cancer. Biomark. Res..

[55] Boudjadi S (2017). Involvement of the integrin alpha1beta1 in the progression of colorectal cancer. Cancers (Basel).

[56] Ibaragi S (2011). Induction of MMP-13 expression in bone-metastasizing cancer cells by type I collagen through integrin alpha1beta1 and alpha2beta1-p38 MAPK signaling. Anticancer Res..

[57] Sottnik JL (2013). Integrin alpha2beta1 (α2β1) promotes prostate cancer skeletal metastasis. Clin. Exp. Metastasis.

