# Supplementary material for: The effects of inhibition and siRNA knockdown of collagen-binding integrins on human umbilical vein endothelial cell migration and tube formation
Source: Sci Rep. 2022 Dec 14;12:21601. doi: 10.1038/s41598-022-25937-1 (PMC9751114; doi:10.1038/s41598-022-25937-1)
Supplement: Supplementary file 1 — Supplementary Figures. [file 41598_2022_25937_MOESM1_ESM.docx]

Supporting data


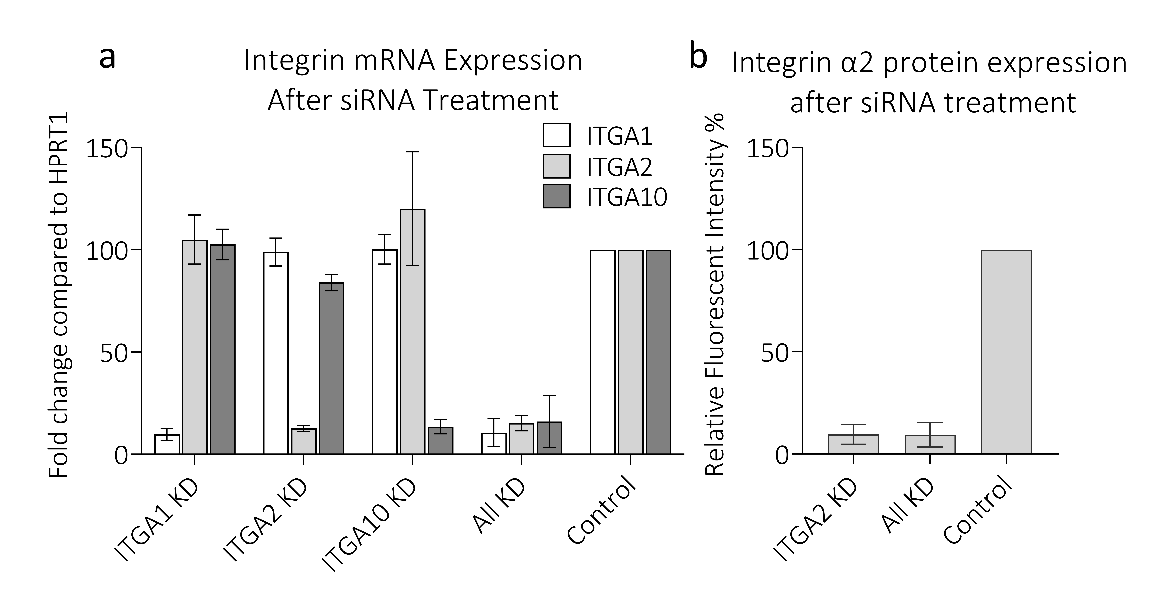

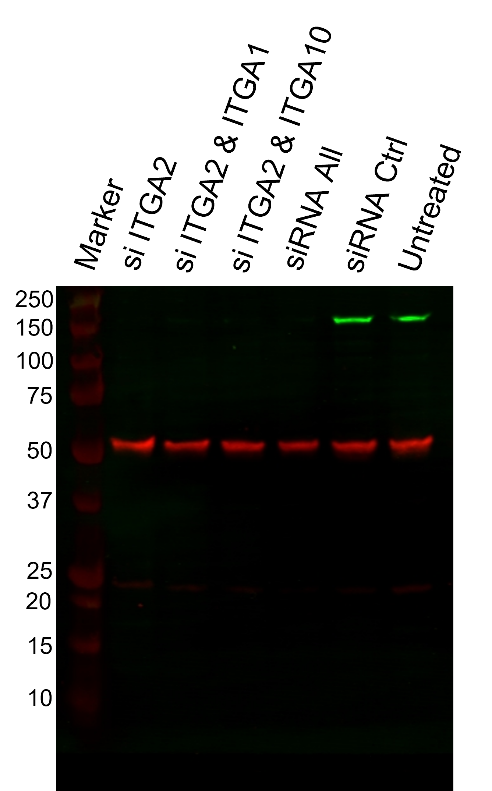


c

Figure S1: siRNA results in 90% knockdown

**Fig S1** Validation of the siRNA knockdown. Silencer Select siRNAs were used to transiently silence the expression of each integrin present, ITGA1, ITGA2 and ITGA10 refer to the siRNA targeting the α1, α2 and α10 subunits, respectively, and finally, All refers to the triple knockdown of all three α subunits together. A) qPCR data showing that the mRNA expression for each integrin was approximately 85-90% lower after target siRNA treatment than with the negative control siRNA. 48 hours after siRNA treatment ITGA1 expression was 9.7 ± 1.7% and 10.6 ± 3.9%, ITGA2 expression was 12.6 ± 0.8%, and 15.2 ± 2.1%, and ITGA10 expression was 13.5 ± 2.0% and 16.0 ± 7.4% in the single and triple knockdowns respectively. The mRNA expression of each integrin after siRNA knockdown is shown as a percentage of the negative control siRNA condition, calculated using the ∆∆CT method. b) Quantification of c) a western blot analysed using the LI-COR system, α2β1 protein was approximately 90% lower after 48 hours siRNA treatment than the control (9.6% ± 4.8% for the single knockdown and 9.5% ± 6.0% for the triple knockdown). This shows the siRNA knockdown is efficient and effective. Relative fluorescence of the α2 subunit (approx. 200kDa green) was normalised to the relative fluorescence of α-tubulin (approx. 50kDa red) for that lane and shown as a percentage of the negative control siRNA condition. Error bars indicate SEM calculated from three repeats

Figure S2: Effects of Integrin inhibition on cell spreading – images


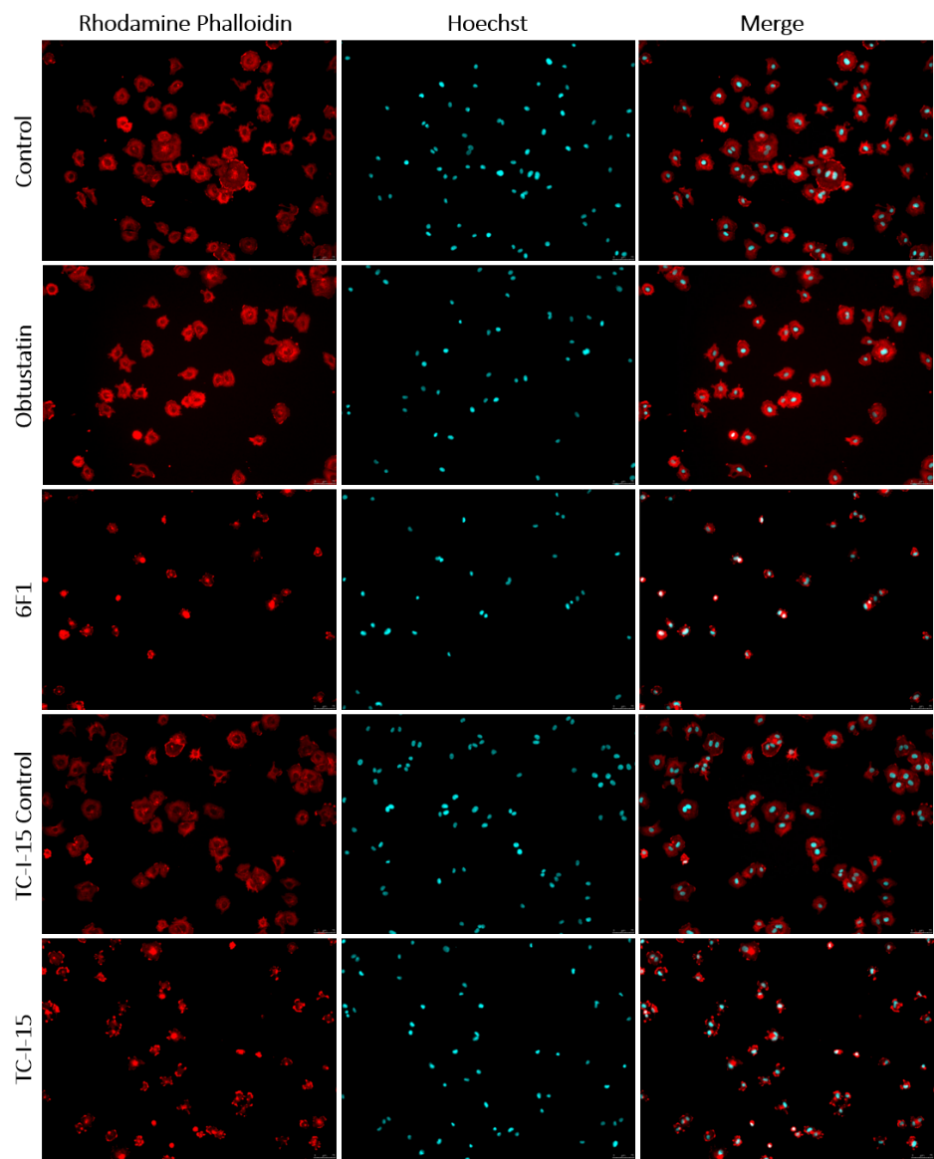
**Fig S2**

**Fig S2** Quantification of cell spreading after integrin inhibition. Fluorescence microscopy images of HUVECs 1 hour after seeding on collagen I coated surfaces in the presence or absence of integrin inhibitors. Cells were fixed with 4% PFA, permeabilised with 0.5% Triton X100 and stained with Hoechst (blue) and Rhodamine Phalloidin (red). Images are taken at 10x magnification.

Figure S3: Effects of Integrin siRNA knockdown on cell spreading – images


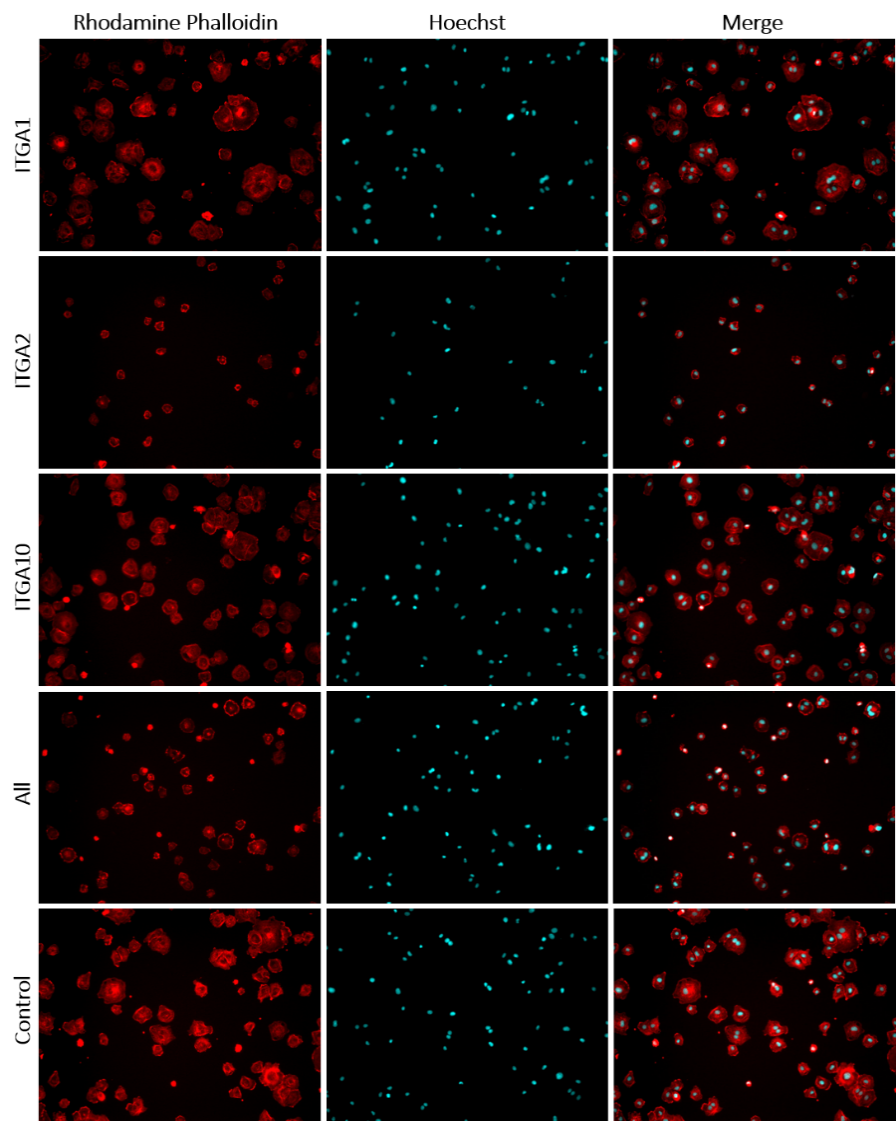


**Fig S3** Quantification of cell spreading after siRNA treatment. Fluorescence microscopy images of HUVECs 1 hour after seeding on collagen I coated surfaces 48-hours post-siRNA treatment. Cells were fixed with 4% PFA, permeabilised with 0.5% Triton X100 and stained with Hoechst (blue) and Rhodamine Phalloidin (red). Images are taken at 10x magnification.


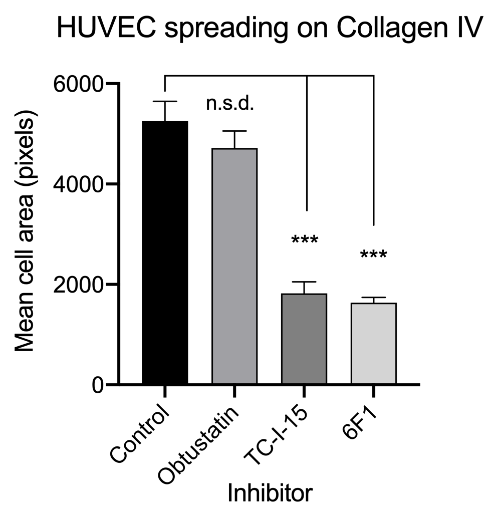
Fig S4 – Effect of integrin inhibition on the spreading of HUVECs on collagen IV

Fig S4 Quantification of HUVEC spreading on collagen IV in the presence of integrin inhibitors. Fluorescence microscopy images of HUVECs 1 hour after seeding on collagen IV coated surfaces in the presence or absence of integrin inhibitors. Cells were fixed with 4% PFA, permeabilised with 0.5% Triton X100 and stained with Hoechst (blue) and Rhodamine Phalloidin (red). Images are taken at 10x magnification. ImageJ was used to quantify the average area per cell (in pixels).

Fig S5 Integrin inhibition or siRNA treatment has no effect on HUVEC proliferation on collagen coated surfaces


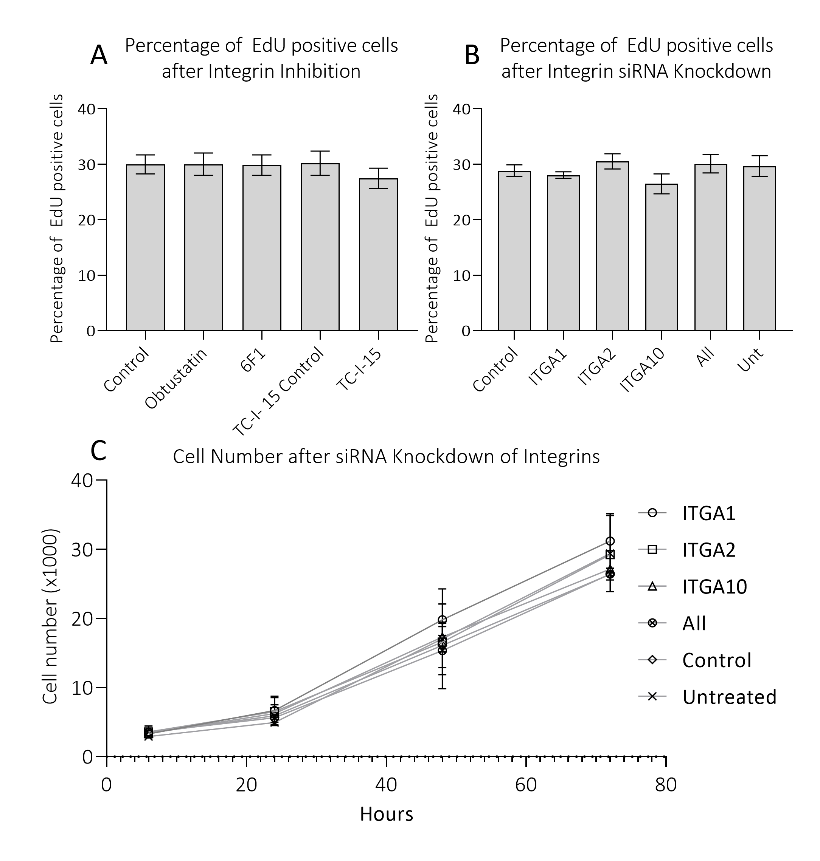


**Fig S5** Graphs A and B show quantification of proliferation as a percentage of EdU positive cells. A) HUVECs were serum starved for 4 hours and then seeded onto collagen I coated surfaces in the presence or absence of inhibitors or vehicle controls. B) HUVECs were serum starved for 4 hours, 48 hours post-siRNA treatment, and then seeded onto collagen coated surfaces. After 24 hours, for A and B, 20µM EdU was added for 2 hours before fixing cells in 4% PFA. Thermo EdU ClickiT kits were used to label EdU, Hoechst was used to label the nucleus. Each condition was carried out in triplicate and repeated three times. 10 fields of view were taken per well. ImageJ was used to quantify the percentage of EdU positive cells. Graph C shows a quantification of cell number after siRNA treatment. HUVECs were seeded onto collagen I coated surfaces 48 hours post-siRNA treatment. At 0, 24, 48 and 72 hours the cell number was quantified using the Roche LDH colorimetric cytotoxicity kit.
